# TDG Gene Polymorphisms and Their Possible Association with Colorectal Cancer: A Case Control Study

**DOI:** 10.1155/2019/7091815

**Published:** 2019-05-23

**Authors:** Narasimha Reddy Parine, Ibrahim O. Alanazi, Jilani Purusottapatnam Shaik, Sooad Aldhaian, Abdulrahman M. Aljebreen, Othman Alharbi, Majid A. Almadi, Nahla A. Azzam, Mohammad Alanazi

**Affiliations:** ^1^Genome Research Chair, Department of Biochemistry, College of Science, King Saud University, Riyadh 11451, Saudi Arabia; ^2^National Center for Biotechnology, King Abdulaziz City for Science and Technology (KACST), P.O. Box 6086, Riyadh 11461, Saudi Arabia; ^3^College of Medicine, King Saud University, Riyadh, Saudi Arabia; ^4^Division of Gastroenterology, King Khalid University Hospital, King Saud University, Riyadh, Saudi Arabia

## Abstract

Genetic alterations that might lead to colorectal cancer involve essential genes including those involved in DNA repair, inclusive of base excision repair (BER).* Thymine DNA glycosylase *(*TDG*) is one of the most well characterized BER genes that catalyzes the removal of thymine moieties from G/T mismatches and is also involved in many cellular functions, such as the regulation of gene expression, transcriptional coactivation, and the control of epigenetic DNA modification. Mutation of the* TDG *gene is implicated in carcinogenesis. In the present study, we aimed to investigate the association between* TDG *gene polymorphisms and their involvement in colon cancer susceptibility. One hundred blood samples were obtained from colorectal cancer patients and healthy controls for the genotyping of seven SNPs in the* TDG* gene. DNA was extracted from the blood, and the polymorphic sites (SNPs) rs4135113, rs4135050, rs4135066, rs3751209, rs1866074, and rs1882018 were investigated using TaqMan genotyping. One of the six* TDG* SNPs was associated with an increased risk of colon cancer. The AA genotype of the* TDG* SNP rs4135113 increased the risk of colon cancer development by more than 3.6-fold, whereas the minor allele A increased the risk by 1.6-fold. It also showed a 5-fold higher risk in patients over the age of 57. SNP rs1866074 showed a significant protective association in CRC patients. The GA genotype of* TDG* rs3751209 was associated with a decreased risk in males. There is a significant relationship between* TDG* gene function and colorectal cancer progression.

## 1. Introduction

The development of cancer is a multistep process involving aberrations in many cellular processes, including differentiation, cell cycle regulation, cell death, proliferation, and genomic conservation due to functional alterations in a variety of genes.* Thymine DNA glycosylase *(*TDG*) is a member of the mismatch uracil glycosylase subfamily. All of these uracil DNA glycosylase (UDG) enzymes have a monofunctional approach of action [1]. UDGs recruit a common base-flipping, DNA intercalation method for substrate identification and catalyze the removal of the N-glycosidic bond of the flipped base, thus creating an abasic site [2]. TDG has a crucial role in DNA repair, particularly BER, in which it specifically identifies G: U and G: T mismatches resulting from the impulsive deamination of 5-methylcytosine. In addition to its DNA repair function, TDG is also involved in other critical cellular processes, such as the regulation of gene expression, transcriptional coactivation, and the regulation of epigenetic DNA modification [3]. TDG has been shown to interact with some transcription factors and especially with nuclear receptors. TDG initiates the BER pathway, which utilizes the base-flipping method to delete the target bases from the DNA forming an AP site. This happens when TDG binds to the promoters of the BER proteins APE, DNA ligase, and Pol *β* [4].

The role of TDG in cancer progression is a hotly debated issue [5]. Its interaction with tumor suppressor P53 (TP53) proteins initially suggested that TDG merely acts as a tumor suppressor. Overexpression of TDG recruits TP53 proteins to the cyclin dependent kinase inhibitor 1A (p21Waf1) gene promoter and increases its transcriptional activity [6]. Moreover, TP53 binding to the TDG promoter will transcriptionally regulate its expression and control the nuclear translocation of TDG [7]. The relationship between TDG and cancer has been studied by a number of research groups who have suggested that genetic variants in* TDG *and other DNA repair genes confer susceptibility to colorectal cancer [8]. Xu and colleagues showed that* TDG* positively regulates the Wnt signaling pathway and is a key driver necessary for the progression of CRC [9]. They also reported that hypermethylation of* TDG* in multiple myeloma cell lines reduced its gene expression. As a result, DNA repair activity became less efficient [10] in pancreatic adenocarcinoma [11]. Finally, a lack of the DNA mismatch repair protein PMS2 (*PMS2) *and reduced* TDG *expression in rectal cancer has been found to produce a supermutator phenotype at CpG sites [12].

Recent studies reported that the SNP rs2888805 (Val367Met) in* TDG* might be implicated in nonmelanoma skin cancer [13]. The* TDG* SNPs rs167715 and rs4135087 might also be associated with the progression of ovarian cancer in most of the BRCA1/2 mutation carriers [14]. The coding region SNP rs369649741 (Arg66Gly) has been reported to be associated with a high risk in familial colorectal cancer patients [8]. Significant associations have been demonstrated between the risk of cancers, including esophageal squamous cell carcinoma and gastric cancer, and the rs4135054 SNP in* TDG* [15]. This study was conducted to determine the association of the DNA repair gene* TDG* SNPs and colon cancer risk in the Saudi population.

## 2. Materials and Methods

### 2.1. Study Population and Patient Selection

The study population was composed of 100 colorectal cancer patients and 192 control subjects from a Saudi population. Patients were recruited from King Saud Medical City. CRC was confirmed via histopathological examination. The age of the CRC cases varied from 21 to 90 years, with a mean age of 61.10 ± 12.17 years. The main exclusion conditions were autoimmune disorders, hereditary nonpolyposis colorectal cancer (HNPCC), or a previous history of any other disorders. CRC patients who had undergone prior chemoradiotherapy were also excluded. A total of 192 controls were recruited. The age of the controls varied from 21 to 87 years with a mean of 57.2 ± 8.34 years. The primary details of the volunteers were collected by a prestructured questionnaire. Each participant was informed in detail about the present study and signed standard consent. The Ethics Committee of King Saud Medical City approved the present study.

### 2.2. Single Nucleotide Polymorphisms (SNPs) Selection, DNA Extraction, and Genotyping

Genomic DNA was extracted from blood samples using a blood DNA kit (QIAGEN DNeasy Blood & Tissue Kit). According to previous reports, six SNPs located in the* TDG* gene were analyzed: rs4135113 (C__31582396_10), rs4135050 (C___1970689_10), rs4135066 (C___1970695_10), rs3751209 (C__11162283_20), rs1866074 (C___3152280_10), and rs1882018 (C__11490839_10). The preliminary data on the SNPs are shown in Table 1. These SNPs were also selected based on literature reviews of SNP associations with various diseases in diverse ethnic groups. The genotyping analysis was conducted using QuantStudio™ 7 Flex Real-Time PCR System (Applied Biosystems) with an endpoint reading of the genotypes [16].

## 3. Results

A total of 100 colorectal cancer patients and 192 normal controls from a Saudi Arabia population were included in the present study. The clinical and the demographic features of the study subjects are described in Supplementary Table1 (Suppl. Table1). Both CRC and normal samples were classified based on demographic parameters such as age and gender. Colorectal cancer samples were further classified based on tumor location, namely, colon or rectum. The average age of the CRC samples was 57.10 ± 12.17 years and of the controls was 58.2 ± 8.34 years.

All six SNPs in the normal control and CRC patient group obeyed Hardy-Weinberg equilibrium (HWE) (Table 1). Table 1 depicts the details of the SNPs used in the present study including the minor allele frequency and the HWE p-value. Out of the six SNPs, two SNPs, rs4135113 and rs1866074, showed a significant association with colorectal cancer. The genotypic distribution of rs4135113 was 75% GG, 18% GA, and 7% AA in colorectal cancer patients and 82% GG, 16% GA, and 2% AA in normal samples. SNP rs4135113 (Gly199Ser) showed a significant risk association with colorectal cancer in Saudi patients for its genotype AA (OR: 3.640, CI: 1.034–12.819, p = 0.03286) (Table 2). The frequency of the minor allele A in patient samples also showed a significant difference compared with that in the healthy controls (OR: 1.675, CI: 1.013–2.769, p = 0.04264) (Table 2).

The genotypic distribution of rs1866074 was 22% AA, 39% AG, and 39% GG in colorectal cancer patients and 12% AA, 43% AG, and 45% GG in the normal samples. The GG allele frequency was low in colorectal cancer patients compared with that in the controls. SNP rs1866074 showed a protective association of the GG allele (OR: 0.501, CI: 0.251–1, p = 0.047) and the additive (AG+GG) allele (OR: 0.51, CI: 0.269–0.964, p = 0.036) (Table 2). The remaining SNPs, rs4135050, rs4135066, rs3751209, and rs1882018, did not show any association with colorectal cancer in the overall analysis (Table 2).

### 3.1. Stratification Analysis

After an overall analysis, we compared the TDG genotype frequencies based on gender. The genotype distributions of male (n = 58) and female (n = 42) patients were compared with those of matched healthy individuals (Tables 3 and 4). Only rs3751209 showed a protective association in female colon cancer patients with the GA genotype (OR, 0.407; CI: 0.196–0.847, p = 0.01495). The heterozygous GA genotype frequency was low in colorectal cancer patients compared with that in the controls (Table 3). No other SNPs showed any significant association with colorectal cancer based on gender (Tables 3 and 4). The frequency of the A allele in patient samples also showed a significant difference compared with that of the healthy individuals (OR: 2.238, CI: 1.059–4.729, p = 0.03159).

**Figure 1:**
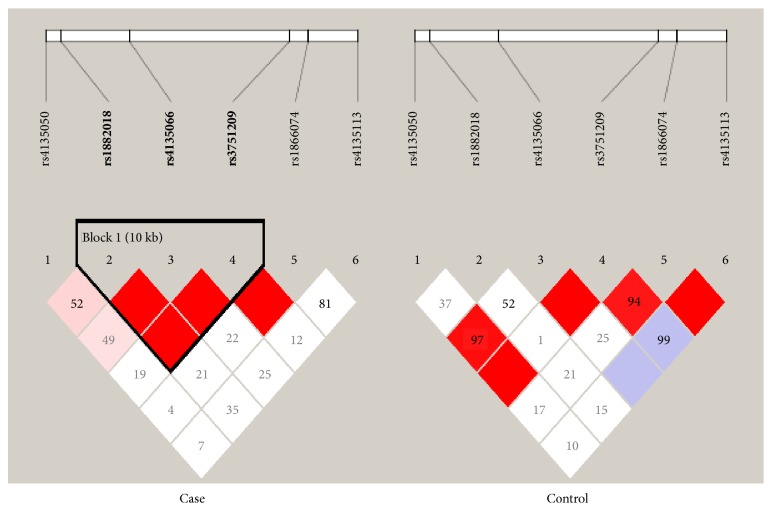
Pairwise LD among the six SNPs in colon cancer and controls. The bright red color indicates a high D′.

The* TDG *genotype distribution was further correlated with the age at colon cancer diagnosis and tumor location. To assess the association of the analyzed SNPs with age at colon cancer diagnosis, we divided the patients into two groups based on the median age of the samples: ≤57 (n = 53) or >57 (n = 47) years of age. The distributions of genotype and allele frequencies for each SNP are shown in Tables 5 and 6. SNP rs4135113, which showed a significant association with CRC in the overall analysis, showed a significant risk association in CRC patients in the group of individuals above 57 years of age. The AA genotype frequency was higher in patients than in healthy individuals. This genotype showed a 5-fold increased risk of colon cancer in the Saudi Arabian population (OR: 5.588; CI: 1.032–30.254; p = 0.02745). In addition to this, the rs4135113 minor allele A also showed a 2-fold increased risk for colorectal cancer in the Saudi population (OR: 2.184, CI: 1.077–4.431; p = 0.02778) (Table 6). A linkage disequilibrium analysis revealed that there was a difference in strength among the SNP associations in cases and controls (Figure 1).

## 4. Discussion

To the best of our knowledge, very few studies have been reported which correlate variation in the* TDG *gene with cancer [16–18]. With the aim of studying the role played by the polymorphisms in the* TDG *gene in CRC risk, we investigated six SNPs (rs4135113, rs4135050, rs4135066, rs3751209, rs1866074, and rs1882018) distributed in different regions of the* TDG *gene. The SNPs were selected based on their location in the* TDG *gene: rs4135113 is located in exon 5; rs4135050, rs4135066, and rs1882018 are in intron 1; and rs3751209 and rs1866074 are in intron 2 and intron 3, respectively. We chose these SNPs to study the effect of mutations in exons and introns. Mutations in an exon might affect the synthesized protein, whereas intron mutations might affect the RNA processing machinery and RNA splicing and stability, which could impact the level of expression and/or protein output [17]. Five of the SNPs were located in intronic region and four of them are in regulatory regions. SNPs rs4135066, rs4135050, and rs1882018 are located in aligned intronic regions flanking alternative conserved exon region (ACE). SNPs rs4135050 and rs1882018 are in exonic splicing silencer (ESS) region, and rs1866074 is in exonic splicing enhancer region.

All six SNPs in the normal control and CRC patient group obeyed the Hardy-Weinberg equilibrium (HWE). Out of the six SNPs, two showed a significant association with CRC. SNP rs4135113 showed a significant risk association of its genotype AA (OR: 3.640, CI: 1.0341–2.819, p = 0.03286) and of the minor allele A (OR: 1.675, CI: 1.013–2.769, p = 0.04264) with colorectal cancer in Saudi patients. The SNP rs1866074 showed a protective association of the GG allele (OR: 0.501, CI: 0.251–1, p = 0.047) and the additive (AG+GG) allele (OR: 0.51, CI: 0.269–0.964, p = 0.036). Our genotyping results showed that there was no association of the other four SNPs (rs4135050, rs4135066, rs3751209, and rs1882018) with CRC patients in the Saudi population in the overall analysis.

The SNP located in the coding region of the* TDG *gene, rs4135113, a G/A transition (missense mutation, Gly199Ser), was studied to detect if there was any association with CRC. There is recent evidence supporting an association between this polymorphism and the development of cancer. Sjolund et al. [15] reported that the Gly199Ser polymorphism occurs in approximately 10% of the global population and the expression of TDG with the G199S variant in human breast epithelial cells might lead to an increased number of DNA double-strand breaks. Thus, it initiates and activates DNA damage that induces cellular transformation and chromosomal aberrations [18]. Our results showed that the A/A genotype variation increases the risk of CRC by approximately fourfold in Saudi patients and is statistically significant (OR= 3.64, p-value = 0.03) (Table 2). Further investigation was conducted to explore the correlation of this polymorphic site with the clinicopathological factors and we observed that rs4135113 showed a fivefold increased risk in old aged patients. A study carried out by Wen-Bin and colleagues (2009) on a Chinese population showed a significant association of rs4135113 with an increased micronucleus in the Chinese population. A few other studies have reported that this SNP has no association with an increased risk of lung cancer, rectal cancer, or gastric adenocarcinoma in a Polish population and a Chinese population [19–21].

We also investigated the effect of rs4135050 on the risk of CRC when the T was substituted by A. The genotype AA in our study showed an elevated CRC risk, although the difference was not statistically significant (Table 2). In an urban Puerto Rican population, the one-carbon nutrient status was not associated with the DNA uracil concentration in this SNP [22].

The SNP rs4135066 has the C substituted by a T. In this investigation, the homozygous TT showed an increased risk of CRC; however, this was not statistically significant (Table 2). A recent study by Barry et al. in an American population showed that the SNP rs4135066 was not statistically associated with prostate cancer [23]. In the rs3751209 polymorphism, the A/G variation in our study showed a reduction in the CRC risk, but the difference did not reach statistical significance (Table 2). A recent study by Osorio et al. showed that this SNP was not associated with breast cancer risk in BRCA1/2 mutation carriers [14]. Another SNP studied was rs1866074, which is located in the intronic region and results from a transition mutation where A is substituted by G. A recent case control study showed that the increase in the frequency of micronuclei in bladder cancer among the AG and GG carriers improved patient prognosis [24]. In this investigation, we observed that the GG genotype and the AG+GG additive genotype decreased the risk of CRC (Table 2). Finally, rs1882018 was studied during this investigation, which is also located in the intronic region and is produced as a result of a transition mutation where A is substituted by G. Our results showed that the GG genotype increased the risk of BC, but the finding did not reach statistical significance (Table 2). A previous study carried out by Wei et al. showed that this SNP had a protective effect against the development of bladder cancer [25].

## 5. Conclusions

In conclusion, the present study showed a significant association between the* TDG* gene and colorectal cancer progression in a Saudi population. One of the six* TDG* SNPs showed an increased risk of colon cancer.* TDG* rs4135113 increased the risk of colon cancer development by more than 3.6- and 1.6-fold in CRC patients in general, and 5-fold in patients aged more than 57 years. SNP rs1866074 showed a significant protective association in CRC patients. The GA genotype of* TDG* rs3751209 showed a decreased risk of CRC in males. Thus, there is a significant relationship between* TDG* gene function and colorectal cancer progression. However, further studies are required to determine the exact effect of amino acid (Gly199Ser) replacement using in vitro methods.

## Figures and Tables

**Table 1:** Primary information for *TDG *polymorphisms.

Genotyped SNP	rs4135113	rs4135050	rs4135066	rs3751209	rs1882018	rs1866074

Chromosome	12	12	12	12	12	12

Chromosome Position	103982915	103968698	103972562	103979822	103969403	103980664

Base change	G>A (Gly199Ser)	T>A	C>T	G>A	C>T	A>G

MAF in our controls	0.10	0.21	0.77	0.31	0.23	0.66

p*-*value for HWE	0.11	0.11	0.09	0.4	0.09	0.52

MAF: minor allele frequency.

HWE: Hardy–Weinberg equilibrium.

**Table 2:** Genotype frequencies of *TDG* gene polymorphism in colorectal cases and controls.

SNP	Variant	Patients Cases	Controls	OR	CI	*χ* ^2^ Value	p-value
rs4135050	TT	58 (0.58)	124 (0.65)	Ref			
	TA	34 (0.34)	55 (0.29)	1.322	0.779–2.244	1.07	0.30107
	AA	8 (0.08)	12 (0.06)	1.425	0.553–3.676	0.54	0.46173
	TA+AA	42 (0.42)	67 (0.35)	1.340	0.816–2.201	1.34	0.24664
	T	150 (0.75)	303 (0.79)	Ref			
	A	50 (0.25	79 (0.21)	1.278	0.853–1.916	1.42	0.23347

rs1882018	CC	58 (0.58)	118 (0.62)	Ref			
	CT	32 (0.32)	59 (0.31)	1.103	0.648–1.880	0.13	0.71725
	TT	10 (0.10)	14 (0.07)	1.453	0.609–3.470	0.71	0.39800
	CT+TT	42 (0.42)	73 (0.38)	1.171	0.715–1.916	0.39	0.53104
	C	148 (0.74)	295 (0.77)	Ref			
	T	52 (0.26)	87 (0.23)	1.191	0.802–1.771	0.75	0.38613

rs4135066	CC	4 (0.04)	14 (0.08)	Ref			
	CT	38 (0.38)	58 (0.30)	2.293	0.702–7.493	1.96	0.16114
	TT	58 (0.58)	119 (0.62)	1.706	0.538–5.413	0.84	0.35998
	CT+TT	96 (0.96)	177 (0.92)	1.898	0.608–5.927	1.25	0.26277
	C	46 (0.23)	86 (0.23)	Ref			
	T	154 (0.77)	296 (0.77)	0.973	0.647–1.462	0.02	0.89402

rs3751209	GG	51 (0.51)	87 (0.46)	Ref			
	GA	38 (0.38)	88 (0.46)	0.737	0.441–1.232	1.36	0.24320
	AA	11 (0.11)	16 (0.08)	1.173	0.505–2.722	0.14	0.71041
	GA+AA	49 (0.49)	104 (0.54)	0.804	0.495–1.305	0.78	0.37654
	G	140 (0.70)	262 (0.69)	Ref			
	A	60 (0.30)	120 (0.31)	0.936	0.645–1.357	0.12	0.72602

rs1866074	AA	22 (0.22)	24 (0.12)	Ref			
	AG	39 (0.39)	82 (0.43)	0.519	0.260–1.037	3.50	0.06152
	GG	39 (0.39)	85 (0.45)	*0.501*	0.251–1.000	3.91	*0.04799*
	AG+GG	78 (0.78)	167 (0.88)	*0.510*	0.269–0.964	4.39	*0.03615*
	A	83 (0.42)	130 (0.34)	Ref			
	G	117 (0.58)	252 (0.66)	0.727	0.511–1.034	3.16	0.07567

rs4135113	GG	75 (0.75)	156 (0.82)	Ref			
	GA	18 (0.18)	31 (0.16)	1.208	0.635–2.297	0.33	0.56458
	AA	7 (0.07)	4 (0.02)	3.640	1.034–12.819	4.55	*0.03286*
	GA+AA	25 (0.25)	35 (0.18)	1.486	0.830–2.660	1.79	0.18130
	G	168 (0.84)	343 (0.90)	Ref			
	A	32 (0.16)	39 (0.10)	1.675	1.013–2.769	4.11	*0.04264*

**Table 3:** Genotype frequencies of *TDG* gene polymorphisms in male colorectal cases and controls.

SNP	Variant	Patients Cases	Controls	OR	CI	*χ* ^2^ Value	p-value
rs4135050	TT	32 (0.55)	60 (0.63)	Ref			
	TA	21 (0.36)	27 (0.28)	1.458	0.714–2.977	1.08	0.29910
	AA	5 (0.09)	8 (0.08)	1.172	0.354–3.879	0.07	0.79493
	TA+AA	26 (0.45)	35 (0.37)	1.393	0.717–2.707	0.96	0.32771
	T	85 (0.73)	147 (0.77)	Ref			
	A	31 (0.27)	43 (0.23)	1.247	0.731–2.126	0.66	0.41728

rs1882018	CC	35 (0.60)	60 (0.63)	Ref			
	CT	18 (0.31)	27 (0.29)	1.143	0.552–2.366	0.13	0.71901
	TT	5 (0.09)	8 (0.08)	1.071	0.325–3.531	0.01	0.90971
	CT+TT	23 (0.40)	35 (0.37)	1.127	0.576–2.204	0.12	0.72787
	C	88 (0.76)	147 (0.77)	Ref			
	T	28 (0.24)	43 (0.23)	1.088	0.631–1.875	0.09	0.76200

rs4135066	CC	3 (0.05)	7 (0.07)	Ref			
	CT	21 (0.36)	30 (0.32)	1.633	0.378–7.054	0.44	0.50827
	TT	34 (0.59)	58 (0.61)	1.368	0.332–5.643	0.19	0.66390
	CT+TT	55 (0.95)	88 (0.93)	1.458	0.362–5.877	0.28	0.59390
	C	27 (0.23)	44 (0.23)	Ref			
	T	89 (0.77)	146 (0.77)	0.993	0.575–1.716	0.001	0.98108

rs3751209	GG	36(0.62)	44 (0.46)	Ref			
	GA	15 (0.26)	45 (0.47)	*0.407*	0.196–0.847	5.92	0.01495
	AA	7 (0.12)	6 (0.07)	1.426	0.440–4.622	0.35	0.55296
	GA+AA	22 (0.38)	51 (0.54)	0.527	0.271–1.027	3.58	0.05840
	G	87 (0.75)	133 (0.70)	Ref			
	A	29 (0.25)	57 (0.30)	0.778	0.461–1.311	0.89	0.34517

rs1866074	AA	12 (0.20)	12 (0.13)	Ref			
	AG	23 (0.40)	40 (0.42)	0.575	0.222–1.487	1.32	0.25137
	GG	23 (0.40)	43 (0.45)	0.535	0.208–1.379	1.70	0.19227
	AG+GG	46 (0.80)	83 (0.87)	0.554	0.230–1.333	1.77	0.18362
	A	47 (0.41)	64 (0.34)	Ref			
	G	69 (0.59)	126 (0.66)	0.746	0.463–1.202	1.45	0.22776

rs4135113	GG	45 (0.78)	75 (0.79)	Ref			
	GA	9 (0.16)	18 (0.19)	0.833	0.345–2.012	0.16	0.68491
	AA	4 (0.07)	2 (0.02)	3.333	0.587–18.937	2.05	0.15266
	GA+AA	13 (0.22)	20 (0.21)	1.083	0.492–2.387	0.04	0.84257
	G	99 (0.85)	168 (0.88)	Ref			
	A	17 (0.15)	22 (0.12)	1.311	0.664–2.588	0.61	0.43370

**Table 4:** Genotype frequencies of *TDG* gene polymorphisms in female colorectal cases and controls.

SNP	Variant	Patients Cases	Controls	OR	CI	*χ* ^2^ Value	p-value
rs4135050	TT	26 (0.62)	64 (0.67)	Ref			
	TA	13 (0.31)	28 (0.29)	1.143	0.513–2.544	0.11	0.74356
	AA	3 (0.07)	4 (0.04)	1.846	0.386–8.828	0.60	0.43682
	TA+AA	16 (0.38)	32 (0.33)	1.231	0.579–2.615	0.29	0.58890
	T	65 (0.77)	156 (0.81)	Ref			
	A	19 (0.23)	36 (0.19)	1.267	0.677–2.370	0.55	0.45905

rs1882018	CC	23 (0.55)	58 (0.60)	Ref			
	CT	14 (0.33)	32 (0.34)	1.103	0.500–2.436	0.06	0.80789
	TT	5 (0.12)	6 (0.06)	2.101	0.584–7.568	1.33	0.24858
	CT+TT	19 (0.45)	38 (0.40)	1.261	0.606–2.623	0.39	0.53475
	C	60 (0.71)	148 (0.77)	Ref			
	T	24 (0.29)	44 (0.23)	1.345	0.753–2.405	1.01	0.31578

rs4135066	CC	1 (0.02)	8 (0.08)	Ref			
	CT	17 (0.40)	28 (0.29)	4.857	0.558–42.3	2.40	0.12134
	TT	24 (0.57)	60 (0.63)	3.200	0.379–26.983	1.26	0.26149
	CT+TT	41 (0.98)	88 (0.92)	3.727	0.451–30.794	1.70	0.19254
	C	19 (0.23)	44 (0.23)	Ref			
	T	65 (0.77)	148 (0.77)	1.017	0.552–1.876	0.0012	0.95677

rs3751209	GG	15 (0.36)	43 (0.45)	Ref			
	GA	23 (0.54)	43 (0.45)	1.533	0.706–3.331	1.17	0.27879
	AA	4 (0.10)	10 (0.10)	1.147	0.313–4.207	0.04	0.83645
	GA+AA	27 (0.64)	53 (0.55)	1.460	0.691–3.087	0.99	0.32021
	G	53 (0.63)	129 (0.67)	Ref			
	A	31 (0.37)	63 (0.33)	1.198	0.701–2.047	0.44	0.50919

rs1866074	AA	10 (0.24)	12 (0.14)	Ref			
	AG	16 (0.38)	42 (0.44)	0.457	0.165–1.265	2.32	0.12761
	GG	16 (0.38)	42 (0.44)	0.457	0.165–1.265	2.32	0.12761
	AG+GG	32 (0.76)	84 (0.88)	0.457	0.180–1.162	2.79	0.09493
	A	36 (0.43)	66 (0.34)	Ref			
	G	48 (0.57)	126 (0.66)	0.698	0.413–1.180	1.80	0.17917

rs4135113	GG	30 (0.71)	81 (0.84)	Ref			
	GA	9 (0.21)	13 (0.14)	1.869	0.725–4.821	1.71	0.19133
	AA	3 (0.07)	2 (0.02)	4.050	0.645–25.439	2.56	0.10991
	GA+AA	12 (0.29)	15 (0.16)	2.160	0.908–5.140	3.11	0.07773
	G	69 (0.82)	175 (0.91)	Ref			
	A	15 (0.18)	17 (0.09)	2.238	1.059–4.729	4.62	0.03159

**Table 5:** Genotype frequencies of *TDG* gene polymorphisms in colorectal cases and controls in the below-57-year-old group.

SNP	Variant	Patients Cases	Controls	OR	CI	*χ* ^2^ Value	p-value
rs4135050	TT	31 (0.58)	67 (0.68)	Ref			
	TA	19 (0.36)	25 (0.25)	1.643	0.789–3.418	1.78	0.18271
	AA	3 (0.06)	7 (0.07)	0.926	0.224–3.824	0.01	0.91567
	TA+AA	22 (0.42)	32 (0.32)	1.486	0.745–2.962	1.27	0.25944
	T	81 (0.76)	159 (0.80)	Ref			
	A	25 (0.24)	39 (0.20)	1.258	0.712–2.223	0.63	0.42813

rs1882018	CC	30 (0.57)	61 (0.62)	Ref			
	CT	17 (0.32)	31 (0.31)	1.115	0.534–2.327	0.08	0.77161
	TT	6 (0.11)	7 (0.07)	1.743	0.538–5.642	0.87	0.34986
	CT+TT	23 (0.43)	38 (0.38)	1.231	0.625–2.423	0.36	0.54797
	C	77 (0.73)	153 (0.77)	Ref			
	T	29 (0.27)	45 (0.23)	1.281	0.745–2.200	0.80	0.36989

rs4135066	CC	2 (0.04)	9 (0.09)	Ref			
	CT	21 (0.40)	31 (0031)	3.048	0.598–15.547	1.93	0.16466
	TT	30 (0.56)	59 (0.60)	2.288	0.465–11.265	1.08	0.29768
	CT+TT	51 (0.96)	90 (0.91)	2.550	0.530–12.260	1.45	0.22791
	C	25 (0.24)	49 (0.25)	Ref			
	T	81 (0.76)	149 (0.75)	1.066	0.613–1.851	0.05	0.82191

rs3751209	GG	26 (0.49)	46 (0.46)	Ref			
	GA	20 (0.38)	44 (0.44)	0.804	0.394–1.643	0.36	0.54978
	AA	7 (0.13)	9 (0.10)	1.376	0.459–4.128	0.33	0.56807
	GA+AA	27 (0.51)	53 (0.54)	0.901	0.462–1.758	0.09	0.76037
	G	72 (0.68)	136 (0.69)	Ref			
	A	34 (0.32)	62 (0.31)	1.036	0.624–1.719	0.02	0.89161

rs1866074	AA	13 (0.25)	13 (0.13)	Ref			
	AG	22 (0.42)	42 (0.42)	0.524	0.208–1.322	1.90	0.16815
	GG	18 (0.33)	44 (0.43)	0.409	0.159–1.052	3.53	0.06029
	AG+GG	40 (0.75)	86 (0.87)	0.465	0.198–1.094	3.16	0.07536
	A	48 (0.45)	68 (0.34)	Ref			
	G	58 (0.55)	130 (0.66)	0.632	0.390–1.023	3.50	0.06132

rs4135113	GG	41 (0.77)	80 (0.81)	Ref			
	GA	10 (0.19)	17 (0.17)	1.148	0.482–2.732	0.10	0.75528
	AA	2 (0.04)	2 (0.02)	1.951	0.265–14.35	0.45	0.50442
	GA+AA	12 (0.23)	19 (0.19)	1.232	0.546–2.784	0.25	0.61496
	G	92 (0.87)	177 (0.89)	Ref			
	A	14 (0.13)	21 (0.11)	1.283	0.623–2.639	0.46	0.49826

**Table 6:** Genotype frequencies of *TDG* gene polymorphisms in colorectal cases and controls in the above-57-year-old group.

SNP	Variant	Patients Cases	Controls	OR	CI	*χ* ^2^ Value	p-value
rs4135050	TT	27 (0.57)	57 (0.62)	Ref			
	TA	15 (0.32)	30 (0.33)	1.056	0.488–2.281	0.02	0.89062
	AA	5 (0.11)	5 (0.05)	2.111	0.563–7.914	1.27	0.25994
	TA+AA	20 (0.43)	35 (0.38)	1.206	0.590–2.466	0.26	0.60699
	T	69 (0.73)	144 (0.78)	Ref			
	A	25 (0.27)	40 (0.22)	1.304	0.733–2.321	0.82	0.36543

rs1882018	CC	28 (0.60)	57 (0.62)	Ref			
	CT	15 (0.32)	28 (0.30)	1.091	0.503–2.363	0.05	0.82605
	TT	4 (0.08)	7 (0.08)	1.163	0.314–4.307	0.05	0.82075
	CT+TT	19 (0.40)	35 (0.38)	1.105	0.539–2.267	0.07	0.78518
	C	71 (0.76)	142 (0.77)	Ref			
	T	23 (0.24)	42 (0.23)	1.095	0.612–1.962	0.09	0.75960

rs4135066	CC	2 (0.04)	5 (0.05)	Ref			
	CT	17 (0.36)	27 (0.29)	1.574	0.274–9.045	0.26	0.60894
	TT	28 (0.60)	60 (0.65)	1.167	0.213–6.387	0.03	0.85883
	CT+TT	45 (0.94)	87 (0.95)	1.293	0.241–6.930	0.09	0.76356
	C	21 (0.22)	37 (0.20)	Ref			
	T	73 (0.78)	147 (0.80)	0.875	0.478–1.602	0.19	0.66485

rs3751209	GG	25 (0.53)	41 (0.45)	Ref			
	GA	18 (0.38)	44(0.47)	0.671	0.320–1.407	1.12	0.28959
	AA	4 (0.09)	7 (0.08)	0.937	0.249–3.527	0.01	0.92351
	GA+AA	22 (0.47)	51 (0.55)	0.707	0.349–1.432	0.93	0.33531
	G	68 (0.72)	126 (0.68)	Ref			
	A	26 (0.28)	58 (0.32)	0.831	0.480–1.438	0.44	0.50706

rs1866074	AA	9 (0.19)	11 (0.12)	Ref			
	AG	17 (0.36)	4 (0.43)	0.519	0.182–1.481	1.52	0.21694
	GG	21 (0.45)	41 (0.45)	0.626	0.224–1.747	0.81	0.36892
	AG+GG	38 (0.81)	81 (0.88)	0.573	0.219–1.500	1.31	0.25305
	A	35 (0.37)	62 (0.34)	Ref			
	G	59 (0.63)	122 (0.66)	0.857	0.510–1.438	0.34	0.55817

rs4135113	GG	34 (0.72)	76 (0.83)	Ref			
	GA	8 (0.17)	14 (0.15)	1.277	0.490–3.33	0.25	0.61607
	AA	5 (0.11)	2 (0.02)	*5.588*	1.032–30.254	4.86	0.02745
	GA+AA	13 (0.28)	16 (0.17)	1.816	0.787–4.191	1.99	0.15870
	G	76 (0.81)	166 (0.90)	Ref			
	A	18 (0.19)	18 (0.10)	*2.184*	1.077–4.431	4.84	0.02778

## Data Availability

The data used to support the findings of this study are available from the corresponding author upon request.

## Supplementary Materials

Supplementary Materials
*Suppl. Table 1*: General clinical parameters of the study participants.
