# Supplementary material for: TDG Gene Polymorphisms and Their Possible Association with Colorectal Cancer: A Case Control Study
Source: J Oncol. 2019 May 23;2019:7091815. doi: 10.1155/2019/7091815 (PMC6556271; doi:10.1155/2019/7091815)
Supplement: Supplementary Materials — Suppl. Table 1: General clinical parameters of the study participants. [file 7091815.f1.docx]

**Supplementary material:**

**Suppl. Table 1:** General clinical parameters of the study participants

| **Variables** | **Cancer**  **N (%)** | **Control**  **N (%)** |
| --- | --- | --- |
| **Participants** | 115 (**100%**) | 192 (**100%**) |
| **Gender** | | |
| Male | 58 (**58%**) | 96 (**50%**) |
| Female | 42 (**42%**) | 96 (**50%**) |
| **Age** | | |
| Below 57 years | 53 (**53%**) | 99 (**51.56%**) |
| Above 57 years | 47 (**47%**) | 93 (**48.43%**) |
| **Median Age (years)** | 57.10 ± 12.17 | 58.2 ± 8.34 |
| **Tumor Localization** | | |
| Colon | 61 (**61%**) | --- |
| Rectum | 39 (**39%**) | --- |
| **Smoking Status** | | |
| Smokers | 7 (**7%**) | 13 (**6.7%**) |
| Nonsmokers | 93 (**93%**) | 179 (**93.3%**) |
